# Heterotopic Ossification in Vertebral Interlaminar/Interspinous Instrumentation: Report of a Case

**DOI:** 10.1155/2012/970642

**Published:** 2012-07-24

**Authors:** Giuseppe Maida, Eleonora Marcati, Silvio Sarubbo

**Affiliations:** Division of Neurosurgery, Department of Neuroscience and Rehabilitation, University-Hospital S. Anna, 8 Via Aldo Moro, 44124 Ferrara, Italy

## Abstract

We present here a rare case of heterotopic ossification in interspinous/interlaminar Coflex device. The classical surgical indications for these implants are degenerative canal stenosis, discogenic low back pain, disk herniations, facet syndrome, and instability. However, fractures of spinous processes are a potential risk after interspinous/interlaminar devices' implantation. Recently, heterotopic ossification, a well-known complication of hip and knee arthroplasty, has been reported after cervical and lumbar prosthesis. We performed undercutting and implantation of the dynamic interspinous/interlaminar device to treat an adult male patient with L4-L5 stenosis. The patient underwent 45-day imaging and clinical followup, and we observed both a neurological and imaging improvement. A CT bone scan, performed 3 years after surgery for recurrence of neurogenic claudication, showed a new stenosis due to an abnormal ossification all over the device. To our knowledge, this is the first reported case of heterotopic ossification in an interspinous/interlaminar dynamic device. Accordingly, we aim to suggest it as a new complication of interspinous/interlaminar devices.

## 1. Introduction

Heterotopic ossification (HO) was defined as an abnormal formation of bone within extraskeletal soft tissues [1, 2]. The pathogenesis of HO is unknown, and it is most commonly seen following total hip and knee arthroplasty [3–9], however, it is also reported in total disc arthroplasties, more frequently in the cervical region [10–20].

We present here what is, to our knowledge, the first reported case of HO secondary to an interspinous/interlaminar dynamic device (Coflex) in an adult male.

## 2. Case Report

### 2.1. History and Examination

**Figure 1:**
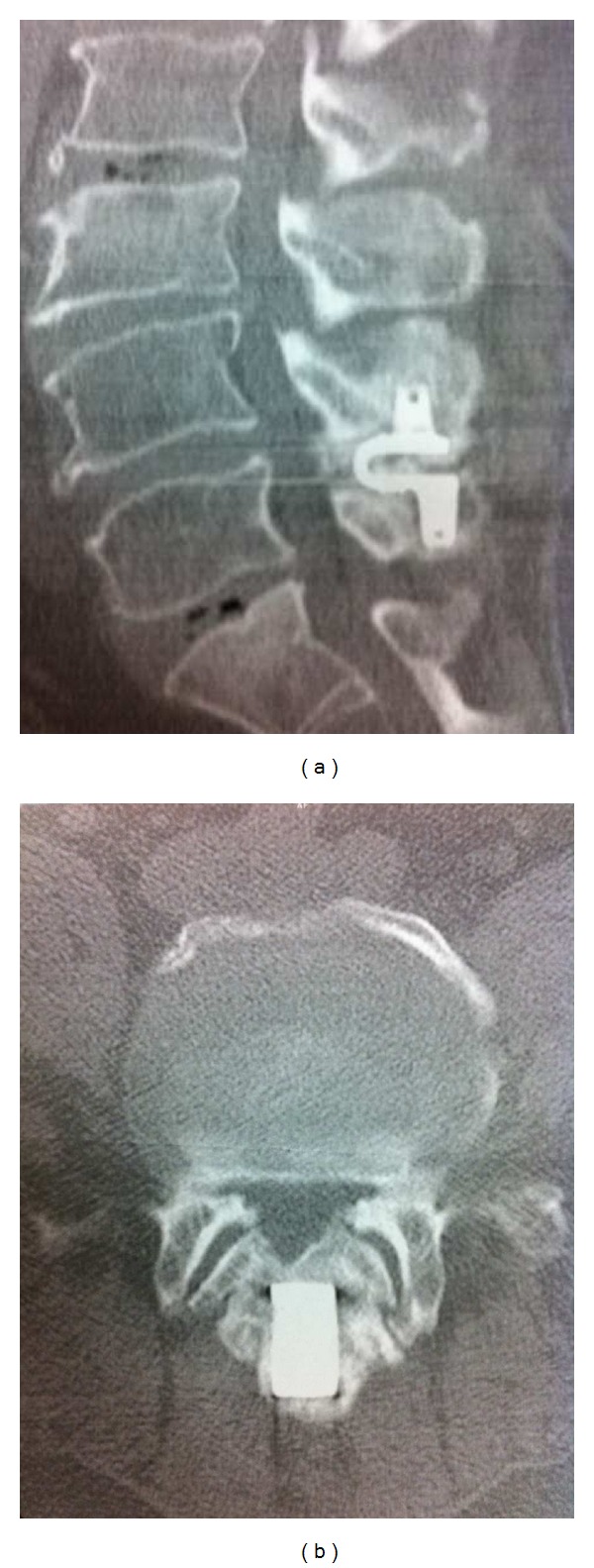
Preoperative CT bone scan showing heterotopic ossification of interspinous/interlaminar Coflex device.

This 58 years-old man presented with a 6-month history of progressively worsening low back pain. The intensity of pain, assessed by using the visual analogue scale (VAS), was scored at 8/10. His pain was radiating to legs and impairing deambulation. His medical history was significant for hypertension. In 2008, the patient underwent microsurgical decompressive undercutting and implantation of an interspinous/interlaminar Coflex device because of L4-L5 stenosis (as documented by MRI).Postoperative and 45-day follow-up radiographic images ensured proper device position and maintenance of the range of motion. On postoperative and 45-day follow-up examination, the patient showed a quite total resolution of the prior clinic (VAS 3/10). In 2011, the patient came to our attention for recurrence of motor weakness with the L5 myotome affected, decreased Achilles and patellar reflexes, and neurogenic claudication. The X-rays, CT scan with bone windows (Figure 1), and MR images revealed a mature ossification of the device with relevant restenosis. Because of the patient's disabling neurological symptoms, it was felt appropriate to treat it surgically.

### 2.2. Operative Technique

The patient was placed in a knee-chest position. A midline incision was then made and soft tissue was accordingly dissected free from the bone in a subperiosteal fashion down to the laminae, on both sides. An abnormal osseous tuberosity was subsequently detected surrounding the L4 and L5 spinous processes. The interspinous/interlaminar Coflex device implanted at that level was not detectable, as also visible in Figure 1. Hence, we started to resect the new bone formation with a chisel. The “U-shaped” part of the device was completely filled by bone. Therefore, we proceeded the resection till the device was free to be removed. In addition, the dura mater was all covered by interlaminar bone which was consequently removed by using Kerrison rongeurs. Finally, gross-total resection of the new bone formation was achieved. Moreover, L4-L5 laminectomy with facet joints preservation and L3, S1 undercutting were performed. Then, secure haemostasis was obtained, and the wound was extensively irrigated and closed in layers. 

### 2.3. Postoperative Course

Resection of the pathologic bone formation resulted in a rapid neurological recovery (VAS 3/10), and the patient could then walk independently. Nonsteroidal antiinflammatory drugs were used for few weeks in the postoperative period. At the 2-month followup, the patient had a great reduction in pain and disability. 

## 3. Discussion

Decompression is a widely accepted intervention for patients with lumbar canal stenosis. When associated with fusion, adjacent segment degeneration may occur. Therefore, dynamic implants like interspinous/interlaminardevices (IDs) were developed [21]. Moreover, lumbar disc rehydration was described after the implantation of posterior dynamic stabilization systems [22]. Several indications have been suggested for interspinous/interlaminar devices, ranging from treatment of degenerative canal stenosis, discogenic low back pain, disk herniations, facet syndrome, and instability [23]. Interspinous/interlaminar devices should bound spinal extension of the treated segment, relieve facet joints and low back pain, and enlarge the spinal canal at the implant level. Besides, these implants allow a less-invasive decompression with a lower rate of complications like cerebrospinal fluid fistula [24]. However, fractures of spinous processes can occur during or afterinterspinousspacer implants, particularly in osteopenic patients [25]. Subsidence of the implant into the bone or dislocation may also be expected [26]. In the literature, several retrospective data on biomechanic, efficacy, and complications of Coflex implants are analysed and described. However, prospective studies concern only a small population, at this time [24, 26–29].

Heterotopic ossification is a widely investigated complication following total hip and knee arthroplasty [3–9]. Recently, it has also been described for cervical [10–20] and lumbar [30, 31] total disc arthroplasty [32, 33] and for posterolateral lumbar spine fusion after the use of bone morphogenetic protein-2 (BMP-2) [34, 35]. HO was defined as an unnatural formation of bone within soft tissues, juxtaposed to the skeleton, and usually not involving the periosteum [1, 2]. HO can result in a variety of complications associated with a decline in the range of motion. It was classified into posttraumatic [36], nontraumatic or neurogenic, and myositis or fibrodysplasia ossificans progressive [1]. The pathogenesis of HO is unknown; an hypothesis is the imbalance in local and systemic factors inducing osteoprogenitor cells [37, 38]. Known risk factors might be the type and size of prosthesis, operative technique, osteoarthritis, injury patterns, male gender, and age [1, 30, 37]. McAfee et al. [39] provided the first classification system of HO after total disc replacement by using five degrees of severity (Table 1), based on the Brooker et al. [40] total hip arthroplasty classification. McAfee's classification depends on flexion-extension X-rays. Nonsteroidal antinflammatory drugs (i.e., indometacin, naproxen, diclofenac, and cyclooxygenase-2 inhibitors) and localized radiotherapy were proposed as a prophylaxis of HO after arthroplasty [41–43]. To treat the acute HO, there is insufficient evidence to recommend some pharmacological agents; hence, to obtain pain relief and to improve the decreased range of motion before complications arise, clinically relevant HO should be resected [44]. The suggested optimal timing of surgery ranges from 12 to 18 months after radiographic evidence of HO maturation [1].

In our case, we chose to resect the HO and to enlarge the prior decompression. Patient's symptoms were quite severe, limiting his daily activities and impairing deambulation. Moreover, the relatively large size of the new bone carried potential risk of a greater neurological damage. Through posterior approach, we performed the resection of the heterotopic bone, removed the device, and achieved a greater decompression. After surgery, a prophylactic treatment with nonsteroidal antiinflammatory drugs was established to prevent the main risk of recurrence.

To our knowledge, the present is the first report of HO after implantation of an interspinous/interlaminar dynamic device.

## 4. Conclusion

The reported case describes the formation of heterotopic ossification with the use of an interspinous/interlaminar Coflex device. These are several recent reported cases of delayed HO after dynamic stabilization systems, but none on interspinous/interlaminar dynamic devices. The effects of the dynamic stabilization systems on disc regeneration have recently been discussed. It is hoped that further studies will evaluate the potential relationship between dynamic stabilization and HO, with or without other known associated risk factors. Above all, the case report's aim is to suggest the possibility of HO as a new complication of interspinous/interlaminar devices.

## Figures and Tables

**Table 1:** McAfee's classification of heterotopic ossification (HO).

McAfee's classification	
(0) No HO	
(I) Islands of bone not within the margins of the disc and not interfering with motion	
(II) Bone within the margins of the disc but not blocking motion	
(III) Bone within the margins of the disc and interfering with motion of the prosthesis	
(IV) Bony ankylosis	

^∗^Adapted from McAfee et al. [39].
